# Health economic evaluation of an electronic mindfulness-based intervention (eMBI) to improve maternal mental health during pregnancy – a randomized controlled trial (RCT)

**DOI:** 10.1186/s13561-024-00537-z

**Published:** 2024-07-30

**Authors:** Lena Hasemann, Svenja Elkenkamp, Mitho Müller, Armin Bauer, Stephanie Wallwiener, Wolfgang Greiner

**Affiliations:** 1 AG 5 - Department of Health Economics and Health Care Management, School of Public Health, Bielefeld University, Universitaetsstrasse 25, 33615 Bielefeld, Germany; 2 Department of Psychology, Ludwig-Maximilians-University of Munich, Leopoldstrasse 13, 80802 Munich, Germany; 3 Department for Women’s Health, Tuebingen University, Calwerstrasse 7, 72076 Tuebingen, Germany; 4 Department of Obstetrics and Gynecology, Heidelberg University, Im Neuenheimer Feld 440, 69115 Heidelberg, Germany

**Keywords:** Pregnancy, Mental health, Depression, Anxiety, Electronic mindfulness-based intervention (eMBI), Health care resource utilization, Health care costs

## Abstract

**Background:**

Anxiety and depression are the most prevalent psychiatric diseases in the peripartum period. They can lead to relevant health consequences for mother and child as well as increased health care resource utilization (HCRU) and related costs. Due to the promising results of mindfulness-based interventions (MBI) and digital health applications in mental health, an electronic MBI on maternal mental health during pregnancy was implemented and assessed in terms of transferability to standard care in Germany. The present study focused the health economic outcomes of the randomized controlled trial (RCT).

**Methods:**

The analysis, adopting a payer’s and a societal perspective, included women of increased emotional distress at < 29 weeks of gestation. We applied inferential statistics (α = 0.05 significance level) to compare the intervention group (IG) and control group (CG) in terms of HCRU and costs. The analysis was primarily based on statutory health insurance claims data which covered the individual observational period of 40 weeks.

**Results:**

Overall, 258 women (IG: 117, CG: 141) were included in the health economic analysis. The results on total health care costs from a payer’s perspective indicated higher costs for the IGi compared to the CG (Exp(ß) = 1.096, 95% CI: 1.006–1.194, *p* = 0.037). However, the estimation was not significant after Bonferroni correction (*p* < 0.006). Even the analysis from a societal perspective as well as sensitivity analyses did not show significant results.

**Conclusions:**

In the present study, the eMBI did neither reduced nor significantly increased health care costs. Further research is needed to generate robust evidence on eMBIs for women suffering from peripartum depression and anxiety.

**Trial registration:**

German Clinical Trials Register: DRKS00017210. Registered on 13 January 2020. Retrospectively registered.

**Supplementary Information:**

The online version contains supplementary material available at 10.1186/s13561-024-00537-z.

## Background

Anxiety and depression are the most prevalent psychiatric diseases in the perinatal period [1, 2]. Meta-analyses and systematic reviews show prevalence rates of 12% and 15% for perinatal depression and anxiety, respectively [1, 3]. In Germany, depressive disorders were observed in approximately 9% and anxiety in 17% of pregnant women [4]. Mental health problems during pregnancy were found to be associated with e.g. postpartum depression, increased cortisol levels, caesarean section, prematurity, low birth weight, poor mother-child-interaction and physiological and impaired (socio-)emotional development in childhood [5–10] as well as increased health care resource utilization (HCRU) and related costs [11, 12]. Bauer et al. [12] account for total lifetime costs of peripartum depression and anxiety amounting to 92,537€ (£75,728 ) and 42,538€ (£34,811) [13] per woman, respectively. These far-reaching consequences for mothers, children, health care systems, and societies highlight the public health relevance of peripartum anxiety and depression.

Due to their efficacy, low costs and low-threshold accessibility, mindfulness-based interventions (MBIs) have become increasingly popular in mental health care [14, 15] and represent a promising approach for women suffering from anxiety and depression during the peripartum period [16–19]. In recent years, digital health has been increasingly prioritized in the German health care system, not least through the promotion of digital health applications [20]. Evidence demonstrates that online-based interventions potentially reduce symptoms of anxiety and depression [21] and appear to be cost-effective [22]. Furthermore, studies indicate that online-based interventions could be a cost-effective way to improve perinatal mental health [23, 24]. However, robust results with a focus on the peripartum period are still lacking.

To address this gap in research, an electronic MBI (eMBI) on maternal mental health during pregnancy was implemented and assessed in terms of transferability to standard care in the German statutory health insurance (SHI). A multicenter, randomized controlled trial (RCT) was conducted to evaluate the effectiveness and the costs of the intervention [25]. Results of the main (effectiveness) evaluation show that the intervention is beneficial, e.g. significantly reduced the risk of postpartum depression [26]. The current study focused on the health economic analysis of the new health care approach and aimed to compare the intervention group (IG) and control group (CG) in terms of total health care costs. Moreover, according to the fact that a high proportion of depression treatments is allocated to general practitioners (GPs) [27], we performed detailed analysis on outpatient HCRU.

## Methods

### Study design

An RCT was performed examining the effectiveness and costs of an eMBI to promote maternal mental health during pregnancy, which was initiated by University women’s hospital of Heidelberg (Germany), implemented in 2018 in cooperation with the Department for Women’s Health at University of Tuebingen and evaluated at Ludwig Maximilian UniversityMunich and Bielefeld University. A full description of the methodology of the RCT can be found elsewhere [25]. In this analysis, we focused on health economic outcomes and compared the IG and the CG in terms of HCRU and costs. The observation period covered 40 weeks, beginning in the last trimester (28th week) of pregnancy. According to methodological guidelines [28], the main analysis adopted a payer’s (SHI) perspective, which was extended to a societal perspective.

### Intervention

The intervention consisted of eight weekly (45-min.) sessions [29, 30] and combined psychoeducation, cognitive behavioural therapy and mindfulness exercises. The eMBI taught women how to deal with stress, anxiety, and depressive symptoms and supported the autonomy of the expectant mother. The digital application included e.g. videos, audio files and interactive worksheets [25].

### Study population

**Fig. 1:**
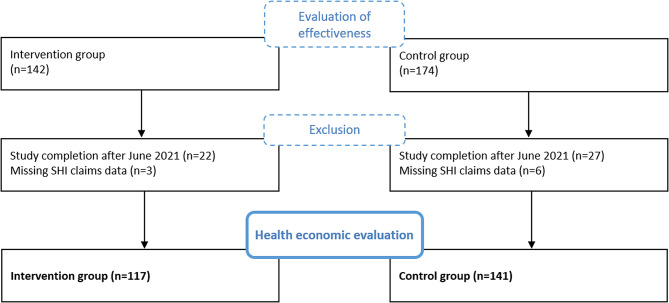
Flow-diagram study population health economic analysis

Study participants were recruited between March 2019 and September 2020 via routine care in the study centers (University Hospitals Heidelberg and Tübingen) and in gynecological practices, where the women were screened routinely for emotional distress. They were eligible to participate in the study if they met the following inclusion criteria: increased level of emotional distress (Edinburgh Postnatal Depression Scale (EPDS) [31, 32] score > 9), age ≥ 18, gestation < 29 weeks, sufficient German language skills, internet access, insured with one of the participating SHI companies. Exclusion criteria were multifetal pregnancy, acute psychotic episodes or psychiatric diagnoses (schizophrenic disorders, suicidality, substance abuse disorders, borderline personality disorder, bipolar disorders, traumatic experiences without reference to the current pregnancy) or the need for an acute psychiatric treatment and participation in a MBI during the current pregnancy. Study participants were randomly assigned at a ratio of 1:1 to the IG (eMBI) or CG (treatment as usual). The group variable was blinded with a binary code and was only known to the study staff and the app developers [25]. Since the claims data were only available for the follow-up period until June 2021, participants with incomplete observation periods were excluded. Thus, the health economic analysis included a subsample of the study population included in the evaluation of the effectiveness (Fig. 1).

### Data collection and outcome measures

The analysis was mainly based on claims data provided by involved SHI companies (Techniker Krankenkasse, AOK Baden-Württemberg, mhplus, GWQ ServicePlus AG). Additionally, self-reported baseline characteristics (e.g. educational level, net household income, number of children at home, level of stress (Patient Health Questionnaire (PHQ) [33, 34]), symptoms of depression (EPDS [31, 32]), pre-existing physical and psychiatric diseases, study center) were included (Additional File1, Table 1). To ensure privacy and data protection, data were pseudonymized and did not allow for re-identification of the individuals. The analysis focused on direct health care costs which include inpatient and outpatient services, pharmaceuticals, therapeutic devices, non-physician specialist services (e.g. physical therapy), university/psychiatric outpatient department services and outpatient surgeries, midwifery services and intervention costs. Besides monetary units, measures of HCRU, such as physician consultations, hospital length of stay (LOS) and daily defined doses (DDD), were reported. The latter were determined by adding data provided by the AOK Research Institute (WIdO) [35, 36]. To broaden the analysis to a societal perspective, productivity losses were added. We followed the friction cost approach, applying a vacancy period of 99 days [37], to avoid overestimations of productivity losses [38]. Indirect costs resulted from the individual’s days of disability documented in SHI claims data multiplied by average values of per day salary ($\:\frac{\text{a}\text{n}\text{n}\text{u}\text{a}\text{l}\:\text{s}\text{a}\text{l}\text{a}\text{r}\text{y}\:\text{i}\text{n}\:\text{G}\text{e}\text{r}\text{m}\text{a}\text{n}\text{y}}{\text{n}\text{u}\text{m}\text{b}\text{e}\text{r}\:\text{o}\text{f}\:\text{e}\text{m}\text{p}\text{l}\text{o}\text{y}\text{e}\text{e}\text{s}}\text{*}365\:\text{d}\text{a}\text{y}\text{s}$) of the general population [38–40].

### Statistical analysis

Baseline characteristics of the study population and outcome variables were examined by descriptive and inferential statistics (e.g. t-tests, Wilcoxon-Mann-Whitney test, chi-square tests). Additionally, Little’s test was conducted to test the assumption of missing completely at random [41]. To analyse the intervention effect on outpatient services and total health care costs, we used generalized additive models [42]. Different model specifications have been investigated to achieve an acceptable model fit. While an inverse gamma distribution addressed the skewness of cost data, a negative binomial distribution fitted the count data (physician consultations). Variable selection strategies were applied to identify potentially relevant independent variables from the data set. Thus, initial model formulas included study group, study center, educational level, number of children at home, stress level (PHQ), symptoms of depression (EPDS) and pre-existing physical and psychiatric diseases as well as interaction terms. The final model formula resulted from backward selection based on the Akaike Information Criterion (AIC) [43]. Following the practical advices on variable selection using AIC defined by Sutherland et al. (2023) [44], 85% as well as 95% confidence Inervals (CI) were calculated. Both, initial and final formulas can be found in Additional File 1 (Table 2). In addition, Bonferroni correction was used to consider multiple testing and was reported for relevant estimations of the regression analyses. Model fit and reliability of the results were examined by diagnostic plots (e.g. residual plot). Sensitivity analyses of total health care costs (SHI-perspective) considered 30% higher and lower intervention costs, as well as an extended sample to address exclusions due to limited data availability. The latter included participants with only slightly shorter (four weeks) observation periods. Statistical analyses were performed using the open-source software R (version 4.2.1) [45] (e.g. Package GAMLSS [42]) and were based on a significance level of α = 0.05.

## Results

As the underlying data differed from the evaluation of the effectiveness, the health economic analysis involved a reduced sample of 258 women (IG: 117,CG: 141) (Fig. 1). The onset of COVID-19 pandemic in 2020 lead to decreasing HCRU in Germany [46, 47]. Thus, we examined the overlapping time span of the pandemic and women’s individual observation period, but found no significant between group differences (*p*-value = 0.877).

The average age of women participating in the study was 32 years and their household income was usually above 1500€ (Table 1). The proportion of women with children at home was non-significantly higher in the IG compared to the CG. A statistically significant difference occurred in the level of education (*p* = 0.005). Further, IG participants (6.30, SD = 2.81) showed significantly lower baseline stress level (PHQ) compared to the CG (7.26, SD = 3.77) (t = 2.269, *p* = 0.024). Little’s test [41] confirmed the assumption of missing completely at random (X²=35.77, *p* = 0.573).

**Table 1:** Baseline characteristics of the study sample

	Intervention (*n* = 117)	Control (*n* = 141)	*p*-value
**Age (n = 258)**	32.33 (SD = 4.51)	32.35 (SD = 4.14)	0.979
**Educational level (n = 236)**			
No school leaving qualification	0 (0.00%)	0 (0.00%)	0.005*
Low secondary qualification	6 (5.88%)	7 (5.22%)	
High secondary qualification	38 (37.25%)	25 (18.66%)	
University of applied sciences entrance qualification	14 (13.73%)	22 (16.42%)	
University entrance qualification	44 (43.14%)	80 (59.70%)	
**Household netto income (n = 227)**			
< 1500 €	27 (28.42%)	32 (24.24%)	0.248
1500–2999 €	45 (47.37%)	58 (43.94%)	
3000–4999 €	17 (17.89%)	32 (24.24%)	
5000–8000 €	6 (6.32%)	10 (7.58%)	
**Number of children at home (n = 236)**			
0	49 (48.51%)	82 (60.74%)	0.060
1	40 (39.60%)	42 (31.11%)	
2	11 (10.89%)	10 (7.41%)	
3 or more	1 (0.99%)	1 (0.74%)	
**Stress level (PHQ) (n = 258)**	6.30 (SD = 2.81)	7.26 (SD = 3.77)	0.024*
**Depressive symptoms (EPDS) (n = 258)**	14.14 (SD = 3.49)	14.01 (SD = 3.40)	0.776
**Pre-existing psychiatric disease (n = 258)**			
No	68 (58.12%)	81 (57.45%)	1.000
Yes	49 (41.88%)	60 (42.55%)	
**Pre-existing physical disease (n = 258)**			
No	54 (46.15%)	60 (42.55%)	0.650
Yes	63 (53.85%)	81 (57.45%)	
**Study center (n = 258)**			
Heidelberg	57 (48.72%)	59 (41.84%)	0.327
Tuebingen	60 (51.28%)	82 (58.16%)	

*SD = standard deviation*, *significance level (p < 0.05)

The main analysis from a health economic perspective focused on total HCRU and costs. Table 2 shows the mean values and standard deviations of women’s resource use within the study period, adopting a SHI-perspective. Most cost-intensive were inpatient services, which were mainly related to childbirth. On average, these costs arose to the amount of 3363.67€ (SD = 1643.36€) in IG and 3509.82€ (SD = 2692.00€) in CG. Further relevant components in terms of costs were outpatient (IG: 1475.06€, SD = 1077.06€; CG: 1491.34€, SD = 1065.37€) and midwifery services (IG: 1156.71€, SD = 834.98€; CG: 1196.95€, SD = 1040.53€). Non-physician services and therapeutic devices generated rather low costs. Wilcoxon-Mann-Whitney tests did not show statistically significant between group differences in utilization or (total) costs of health care services.

**Table 2:** HCRU and costs IG vs. CG

	HCRU			Costs		
	Intervention (*n* = 117)	Control (*n* = 141)	*p*-value	Intervention (*n* = 117)	Control (*n* = 141)	*p*-value
**Outpatient Services** Consultations/€	30.74 (SD = 15.73)	31.30 (SD = 16.70)	0.831	1475.06 (SD = 1077.06)	1491.34 (SD = 1065.37)	0.978
**Inpatient Services** LOS/€	5.30 (SD = 3.75)	6.45 (SD = 8.95)	0.634	3363.67 (SD = 1643.36)	3509.82 (SD = 2692.00)	0.443
**Pharmaceuticals** DDD/€	174.95 (SD = 259.08)	159.07 (SD = 228.00)	0.900	125.56 (SD = 210.85)	190.92 (SD = 641.98)	0.520
**Univ./psych. outpatient department**,** outpatient surgeries** Cases/€	0.59 (SD = 0.98)	0.59 (SD = 1.04)	0.966	110.99 (SD = 237.91)	108.15 (SD = 207.83)	0.992
**Therapeutic devices** Items/€	1.32 (SD = 1.96)	1.70 (SD = 2.43)	0.390	65.65 (SD = 152.81)	121.77 (SD = 495.83)	0.280
**Non-physician specialist services** Items/€	0.69 (SD = 1.53)	0.89 (SD = 2.55)	0.642	49.00 (SD = 99.75)	73.26 (SD = 256.69)	0.649
**Midwifery services** Days/€	201.49 (SD = 114.05)	225.35 (SD = 105.23)	0.103	1156.71 (SD = 834.98)	1196.95 (SD = 1040.53)	0.987
**Intervention (eMBI)** Items/€	1	NA	NA	216.86 (+/- 30%)	NA	NA
**Total health care costs (SHI-perspective) (€)**				6563.49 (SD = 2474.05)	6692.21 (SD = 3608.22)	0.112

*SD = standard deviation*, *significance level (p < 0.05)

The generalized additive model (inverse gamma distribution) on total health care costs from a SHI-perspective (Table 3) indicated higher costs for women in the IG compared to the CG (Exp(ß) = 1.096, 95%-CI: 1.006–1.194, *p* = 0.037). However, the estimate was not significant after Bonferroni correction (new significance level *p* < 0.006). Besides the study group, the final regression term included the number of children at home, initial stress level and pre-existing psychiatric and physical diseases. Considering the 85% CI does not lead to opposing interpretations of the model (Additional File 1, Table 3). According to model diagnostics, the model fit showed deviations, particularly at lower and upper margins. Residual and QQ-plots did not show further inaccuracies (Additional File 1, Figs. 1–2). Sensitivity analyses, considering 30% higher and lower intervention costs and an extended study population, differed only slightly from the main results and did not show a significant intervention effect after Bonferroni correction (*p* < 0.006) (Additional File 1, Tables 5 and 6).

**Table 3:** Generalized additive model (inverse gamma) - health care costs (main analysis, SHI-perspective)

	Exp(ß)	95% CI	p-value *(unadjusted)*
Intercept	5083.530	4509.622, 5730.476	< 0.001*
Study group (IG)	1.096	1.006, 1.194	0.037*
Number of children at home (1)	0.795	0.725, 0.871	< 0.001*
Number of children at home (2)	0.845	0.727, 0.982	0.029*
Number of children at home (3)	0.691	0.437, 1.092	0.115
Stress level (PHQ)	1.017	1.004, 1.031	0.011*
Pre-existing psychiatric disease (Yes)	1.082	0.991, 1.182	0.082
Pre-existing physical disease (Yes)	0.906	0.833, 0.985	0.021*

*significance level (p < 0.05)

*Global Deviance: 4264.767*

*AIC: 4282.767*

*SBC: 4313.941*

*N* = 236

*Degrees of freedom: 9*

*Residual degrees of freedom: 227*

Productivity losses were measured by women’s days of disability and the mean value of salary of the general population. The durations of disability did not significantly differ between IG (1.37, SD = 4.47) and CG (1.38, SD = 6.07) (*p* = 0.211). They did not lead to differences in productivity losses (IG: 169.40€, SD = 553.92€; CG: 170.55€, SD = 751.67€; *p* = 0.213) or total health care costs from a societal perspective, which were 6732.89€ (SD = 2646.74€) in the IG and 6862.76€ (SD = 3801.57€) in the CG (*p* = 0.082). Along with the analysis adopting a SHI-perspective, we found no statistically significant intervention effect (Exp(ß) = 1.102, 95% CI: 1.008–1.204, *p* = 0.033) after Bonferroni correction (*p* < 0.006) on total health care costs from a societal perspective (Table 4). The model diagnostics did not show recognizable differences from the main analysis (Additional File 1, Figs. 3 and 4).

**Table 4:** Generalized additive model (inverse gamma) - health care costs (societal perspective)

	Exp(ß)	95% CI	p-value *(unadjusted)*
Intercept	5098.100	4505.001, 5769.283	< 0.001*
Study group (IG)	1.102	1.008, 1.204	0.033*
Number of children at home (1)	0.806	0.733, 0.887	< 0.001*
Number of children at home (2)	0.853	0.73, 0.996	0.046*
Number of children at home (3)	0.684	0.426, 1.098	0.117
Stress level (PHQ)	1.017	1.003, 1.031	0.015*
Pre-existing psychiatric disease (Yes)	1.084	0.989, 1.188	0.085
Pre-existing physical disease (Yes)	0.904	0.829, 0.986	0.023*

*significance level (p < 0.05)

*Global deviance: 4291.148*

*AIC: 4309.148*

*SBC: 4340.322*

*N* = 236

*Degrees of freedom: 9*

*Residual degrees of freedom: 227*

Besides total HCRU, details on outpatient service utilization were analysed. Women included in the study utilized at least one outpatient service within the observed period. On average the IG had 30.74 and CG participants had 31.30 consultations (Table 5), leading to costs of 1475.06€ and 1491.34€, respectively. Wilcoxon-Mann-Whitney tests did not reveal significant differences between IG and CG in terms of consultations (*p* = 0.831) and outpatient costs (*p* = 0.978). Even the differentiated analyses did not show any significant between group differences in terms of GP and specialist consultations or costs. Whereas women who participated in the intervention had on average 5.71, CG participants had 5.18 GP consultations. As expected, gynaecological and obstetric services represented the most relevant specialist care in terms of utilization and costs (IG: 13.22, 728.58€; CG: 13.28, 718.52€). The number of specialist consultations in neurology, psychiatry or psychotherapy was non-significantly lower in IG (3.01) compared to CG (3.48). These services caused the highest average cost per case in outpatient care. 

**Table 5:** Outpatient services overall and differentiated by GP and specialists

	Intervention (n = 117)	Control (n = 141)	p-Value
Outpatient services			
**Physician consultations**	30.74 (SD = 15.73)	31.30 (SD = 16.70)	0.831
**Costs (€)**	1475.06 (SD = 1.077.06)	1491.34 (SD = 1.065.37)	0.978
**General practitioner services**			
**Physician consultations**	5.71 (SD = 5.46)	5.18 (SD = 5.10)	0.480
**Costs (€)**	185.24 (SD = 184.82)	163.86 (SD = 192.07)	0.269
**Specialist services**: Neurology, Psychiatry, Psychotherapy			
**Physician consultations**	3.01 (SD = 7.12)	3.48 (SD = 8.50)	0.432
**Costs (€)**	284.35 (SD = 712.21)	322.48 (SD = 812.31)	0.343
**Specialist services**: Gynaecology and Obstetrics			
**Physician consultations**	13.22 (SD = 4.71)	13.28 (SD = 5.15)	0.808
**Costs (€)**	728.58 (SD = 282.51)	718.52 (SD = 219.04)	0.736
**Specialist services**: Other			
**Physician consultations**	8.67 (SD = 6.78)	9.26 (SD = 7.24)	0.493
**Costs (€)**	276.87 (SD = 412.70)	286.49 (SD = 448.84)	0.470

*SD = standard deviation*, *significance level (p < 0.05)

Multivariate analyses were performed on the number of overall physicians as well as GP consultations. The results of the generalized additive model (negative binomial distribution) on the total number of physician consultations can be found in Additional File 1 (Table 7). The estimations did not reveal a statistically significant reduction in outpatient HCRU due to the intervention. In contrast, IG participants with a pre-existing psychiatric disease showed an increased number of consultations (Exp(ß) = 1.407, 95% CI: 1.116–1.775, *p* = 0.004), which was significant after Bonferroni correction (*p* < 0.006). Due to this interaction, the interpretation of the study group effect required the consideration of a pre-existing psychiatric disease (Yes/No). In terms of GP consultations (Additional File 1, Table 8), we found no statistically significant intervention effect (Exp(ß) = 1.224, 95% CI: 0.947–1.584, *p* = 0.124). However, diagnostic plots for both regression models on outpatient service utilization accounted for limited quality of the estimations. Thus, the fitted values did not adequately cover the observed values and the results are of limited validity (Additional File, Figs. 5, 6, 7 and 8).

## Discussion

In this study we aimed to evaluate the impact of an eMBI for expectant mothers on HRCU and total costs, adopting a SHI-perspective, extended to a societal perspective. The intervention was not found to cause reductions in total health care costs of women in the third trimester of pregnancy until five months after birth. Analyses of total health care costs adopting a SHI and societal perspective showed non-significantly higher costs for the IG compared to the CG. The sensitivity analyses did not lead to divergent results and interpretations. Moreover, the analyses of outpatient services did not result in conclusions regarding an interventional effect and accounted for limited model quality. The diagnostic plots for the main models were acceptable but showed deviations, particularly at lower and upper margins.

The relevance of digital health applications has considerably increased in recent years. Online interventions are potentially cost-effective alternatives in the treatment of depression and anxiety. However, the conditions and cost components of evaluation studies vary widely [48–50]. The health economic perspective has rarely been considered in previous evaluations of digital interventions for peripartum depression or anxiety disorders [51]. The cost-effectiveness of non-digital approaches has been the subject of numerous studies, but even in this field, the heterogeneity of designs and settings leads to uncertainties [52]. Monteiro et al. (2022) examined a web-based self-directed cognitive behavioural therapy to promote mental health in mothers at low risk for postpartum depression using waitlist comparison. According to the results, the approach leads to non-significant cost savings and a non-significant increase in Quality-Adjusted Life Years (QALYs). The authors conclude that from a societal perspective, implementing the intervention could be a cost-effective approach to improve peripartum mental health [23]. Zheng et al. (2022) compare two psychoeducational interventions (web-based/home-based) with a control group in their three-arm RCT. The study focuses on first-time mothers in the early postpartum period and states that the web-based intervention dominates both alternatives and has the highest probability of cost-effectiveness. However, the researchers point out uncertainties of the analyses. Thus, the present study is consistent with the state of research in terms of non-significant results and existing uncertainties [24].

When interpreting our results, the following strengths and weaknesses should be considered. Compared to the general female population in Germany [53], the study participants represent a quite highly educated sample, with a proportion of about 68% having a university or university of applied sciences entrance qualification. Previous studies showed associations between individual socioeconomic factors and the level of HCRU. As the effect depends on the health care sector [54], the impact on external validity cannot be conclusively assessed. With regard to the percentage of study participants with no children at home (probably primiparous, about 56%), the sample is approximately comparable to the target group in terms of age [55]. Considerable effort was made to generate robust results from the analysis. The study design (RCT) is considered the gold standard in scientific research and prevents several potential biases [56]. In addition, possible confounders and uncertainties were addressed by multivariate analyses and sensitivity analyses. Along with usual standards in RCTs, the sample size calculation of the current study was based on the primary (effectiveness) outcome. It should be noted, that the health economic evaluation could have required a larger sample size to detect significant effects [57]. Although the randomization intends to ensure structural balance of the study groups, it cannot be ruled out that the results might have been affected by latent unmeasured variables (e.g. individual coping mechanisms). Further limitations refer to the process of model selection by AIC which is characterized by its practicability but entails a potential bias due to multiple testing. Thus, inflated significance was addressed by Bonferroni correction. Additionally, along with suggestions on model reporting using AIC [44], 95% and 85% CIs were calculated to provide intervals consistent with the selection strategy.

The analysis was mainly based on SHI claims data, which provide comprehensive and objective information on participants’ HCRU and related costs. However, it should be noted that the data are not primarily collected for research purposes [58, 59]. The heterogeneity of claims data provided by different SHIs requires intensive data preparation [60]. To examine the plausibility of SHI data, reports available from e.g. the Federal Association of SHI Physicians [61] and WIdO [62] were used for comparison and did not refer to implausibility. In general, it can be assumed, that the usage of German SHI claims data will be improved by the Health Data Lab (HDL)[63]. In terms of productivity losses, potential overestimations need to be considered. The calculation was based on national average values. Due to non-gender specific national average values, the comparatively low employment and salary level of women is not represented in the data [64]. In general, indirect costs caused by incapacity for work account for a large proportion of total costs of mental illness [65]. Within the present population this cost component is less relevant (approximately 2.5% of total health care costs from a societal perspective), because of the relatively short observational period of 40 weeks including maternity leave and in many cases parental leave which contradict the status of incapacity for work [66]. In addition, mindfulness interventions during pregnancy might have far-reaching benefits [67]. Due to a restricted study period, potential long-term effects on HCRU and costs could not be examined. Some recommendations for future research can be derived from the limitations. For more valid results, health economic outcomes should be included in power calculations. Another methodological improvement refers to alternative model selection strategies, such as cross-validation. Moreover, studies should be based on comprehensive data sets. These should include potential confounders as well as HCRU and cost data covering extended observation periods. Also, for this purpose, the results generated from the current study could inform predictive health economic modelling approaches.

## Conclusion

In the present study, the eMBI was not found to reduce nor significantly increase health care costs. Overall, these findings are in line with the current state of research, yielding non-significant results. Further research is needed to strengthen the evidence on interventions to promote perinatal mental health in general as well as eMBIs for women suffering from peripartum depression and anxiety. Ideally, the studies should involve women’s long-term impairments and costs as well as the wide-ranging and intergenerational economic consequences.

### Electronic supplementary material

Below is the link to the electronic supplementary material.

Additional File 1

## Data Availability

The datasets analysed for the current study are not publicly available due to project specific agreements of data protection. On reasonable request, possibilities of data access for external researchers have to be proved. Requests can be made to the corresponding author.

## Abbreviations

AIC: akaike information criterion
CEA: cost-effectiveness
CG: control group
CUA: cost-utility analysis
DDD: Defined Daily Dose
eMBI: electronic mindfulness-based intervention
EPDS: Edinburgh Postnatal Depression Scale
GP: general practitioners
HRQoL: health-related quality of life
LOS: length of stay
PHQ: Patient Health Questionnaire
IG: intervention group
RCT: randomized controlled trial
SD: standard deviation
SHI: Statutory health insurance
WIdO: AOK Research Institute
QALY: Quality-Adjusted Life Years
