# Supplementary material for: Health economic evaluation of an electronic mindfulness-based intervention (eMBI) to improve maternal mental health during pregnancy – a randomized controlled trial (RCT)
Source: Health Econ Rev. 2024 Jul 30;14:60. doi: 10.1186/s13561-024-00537-z (PMC11290259; doi:10.1186/s13561-024-00537-z)
Supplement: Supplementary file 1 — Additional File 1 [file 13561_2024_537_MOESM1_ESM.pdf]

Health economic evaluation of an electronic mindfulness-based intervention (eMBI) to improve maternal mental health during pregnancy – a randomized controlled trial (RCT)

Lena Hasemann, Svenja Elkenkamp, Mitho Müller, Armin Bauer, Stephanie Wallwiener, Wolfgang Greiner

Corresponding author: Lena Hasemann, AG 5 - Department of Health Economics and Health Care Management, Bielefeld University, School of Public Health, Universitaetsstrasse 25, 33615 Bielefeld, Germany, E-Mail: lena.hasemann@uni-bielefeld.de

Additional File 1:

1. Overview on variables and analyses

Table 1: Overview of variables, measurements and analyses

Variable	Measurement	Analysis
**Baseline Characteristics**		
**Age**	Years at enrolment	t-Test
**Educational level**	No formal education, elementary school, secondary school, technical diploma, diploma	Wilcoxon-Mann-Whitney-U-Test
**Net household income**	<1500€; 1500-2999€; 3000-4999€; 5000-8000€	Wilcoxon-Mann-Whitney-U-Test
**Number of children at home**	0; 1; 2; 3	Wilcoxon-Mann-Whitney-U-Test
**Stress level**	PHQ-Stress-Scale (0-20)	t-Test
**Symptoms of Depression**	EPDS Score (0-30)	t-Test
**Pre-existing psychiatric disease**	Yes/No	X^2-Test
**Pre-existing physical disease**	Yes/No	X^2-Test
**Study center**	Heidelberg/Tuebingen	X^2-Test
**Health care utilisation/ costs**		
**Overall health care costs (Payer's-/ SHI-perspective)**	Costs (€) including: inpatient services, outpatient services, pharmaceuticals, therapeutic devices, non-physician specialist services (e.g. physical therapy), university/psychiatric outpatient department services and outpatient surgeries, midwives' services, intervention costs (IG)	Wilcoxon-Mann-Whitney-U-Test<br><br>GAM
**Overall health care costs (Societal perspective)**	Costs (€) including: inpatient services, outpatient services, pharmaceuticals, therapeutic devices, non-physician specialist services (e.g. physical therapy), university/psychiatric outpatient department services and outpatient surgeries, midwives' services, intervention costs (IG), productivity losses	
**Outpatient services, General practitioner services,**	Consultations	Wilcoxon-Mann-

**Specialist services** (Neurology, Psychiatry, Psychotherapy; Gynaecology and Obstetrics; Other)		Whitney-U-Test<br><br>GAM
	Costs (€)	Wilcoxon-Mann-Whitney-U-Test
	Costs per case (€)	
**Inpatient services**	Hospital admissions	
	Length of stay (LOS)	Wilcoxon-Mann-Whitney-U-Test
	Costs (€)	
	Costs per case (€)	
**Pharmaceuticals**	Prescriptions	
	DDDs	Wilcoxon-Mann-Whitney-U-Test
	Costs (€)	
	Costs per prescription (€)	Wilcoxon-Mann-Whitney-U-Test
**University/psychiatric outpatient department services and outpatient surgeries**	Services	
	Costs (€)	
**Therapeutic devices**	Items on prescription	Wilcoxon-Mann-Whitney-U-Test
	Costs (€)	
**Non-physician specialist services**	Items on prescription	
	Costs (€)	
**Midwivery services**	Days	
	Costs (€)	
**Productivity losses**	Disability days	
	Costs (€)	

2. Model selection

Table 2: Model selection: Initial and final formulas

**Total health care costs (payer's/ SHI-perspective and societal perspective)**	
Initial model	$total\_health\_care\_costs(direct)_i = \beta_0 + \beta_1*study\_group_i + \beta_2*study\_center_i$<br>$+ \beta_3*no\_children\_at\_home_i + \beta_4*stress\_level_i + \beta_5*symptoms\_depr_i$<br>$+ \beta_6*pre\_psych\_disease_i + \beta_7* pre\_phys\_disease_i + \beta_8*study\_group_i*study\_center_i$<br>$+ \beta_9*study\_group_i*pre\_phys\_disease_i + \beta_{10}*study\_center_i*pre\_phys\_disease_i$<br>$+ \beta_{11}*study\_group_i *study\_center_i *pre\_phys\_disease_i + \varepsilon_i$
Final model	$total\_health\_care\_costs_i = \beta_0 + \beta_1*study\_group_i + \beta_2*no\_children\_at\_home_i +$<br>$\beta_3*stress\_level_i + \beta_4*pre\_psych\_disease_i + \beta_5*pre\_phys\_disease_i + \varepsilon_i$
**Physician consultations (overall)**	
Initial model	$Physician\_consultations_i = \beta_0 + \beta_1*study\_group_i + \beta_2*study\_center_i$<br>$+ \beta_3*no\_children\_at\_home_i + \beta_4*stress\_level_i + \beta_5*symptoms\_depr_i$<br>$+ \beta_6*pre\_psych\_disease_i + \beta_7* pre\_phys\_disease_{i+} + \beta_8*study\_group_i*study\_center_i$<br>$+ \beta_9*study\_group_i*pre\_psych\_disease_i + \beta_{10}*study\_center_i* pre\_psych\_disease_i$<br>$+ \beta_{11}*study\_group_i*study\_center_i*pre\_psych\_disease_i + \varepsilon_i$
Final model	$Physician\_consultations_i = \beta_0 + \beta_1*study\_group_i + \beta_2*no\_children\_at\_home_i$<br>$+ \beta_3*stress\_level_i + \beta_4*pre\_psych\_disease_i + \beta_5* pre\_phys\_disease_i$<br>$+ \beta_6*study\_group_i*pre\_psych\_disease_i + \varepsilon_i$
**General practitioner consultations**	
Initial model	$GP\_consultations_i = \beta_0 + \beta_1*study\_group_i + \beta_2*study\_center_i + \beta_3*no\_children\_at\_home_i$<br>$+ \beta_4*educational\_level_i + \beta_5*stress\_level_i + \beta_6*symptoms\_depr_i + \beta_7*pre\_psych\_disease_i$<br>$+ \beta_8* pre\_phys\_disease_i + \beta_9*study\_group_i*study\_center_i$<br>$+ \beta_{10}*study\_group_i*pre\_phys\_disease_i + \beta_{11}*study\_center_i*pre\_phys\_disease_i$<br>$+ \beta_{12}*study\_group_i *study\_center_i *pre\_phys\_disease_i + \varepsilon_i$
Final model	$GP\_consultations_i = \beta_0 + \beta_1*study\_group_i + \beta_2*no\_children\_at\_home_i$<br>$+ \beta_3*educational\_level_i + \beta_4*stress\_level_i + \beta_5* pre\_phys\_disease_i + \varepsilon_i$

3. Multivariate analysis of total health care costs: Add on 85% CI

Table 3: Generalized additive model (inverse gamma) - health care costs (main analysis, SHI-perspective) incl. 85% CI

	**Exp(ß)**	**85 % CI**	**95 % CI**	**p-value** *(unadjusted)*
Intercept	5083.530	4655.373, 5551.065	4509.622, 5730.476	<0.001*
Study group (IG)	1.096	1.029, 1.167	1.006, 1.194	0.037*
Number of children at home (1)	0.795	0.742, 0.850	0.725, 0.871	<0.001*
Number of children at home (2)	0.845	0.757, 0.943	0.727, 0.982	0.029*
Number of children at home (3)	0.691	0.494, 0.967	0.437, 1.092	0.115
Stress level (PHQ)	1.017	1.008, 1.027	1.004, 1.031	0.011*
Pre-existing psychiatric disease (Yes)	1.082	1.014, 1.154	0.991, 1.182	0.082
Pre-existing physical disease (Yes)	0.906	0.852, 0.963	0.833, 0.985	0.021*

**significance level (p<0.05)*
Global Deviance: 4264.767
AIC: 4282.767
SBC: 4313.941
N=236
Degrees of freedom: 9
Residual degrees of freedom: 227

Table 4: Generalized additive model (inverse gamma) - health care costs (societal perspective) incl. 85% CI

	**Exp(ß)**	**85 % CI**	**95 % CI**	**p-value** *(unadjusted)*
Intercept	5098.100	4655.406, 5582.892	4505.001, 5769.283	<0.001*
Study group (IG)	1.102	1.032, 1.176	1.008, 1.204	0.033*
Number of children at home (1)	0.806	0.752, 0.864	0.733, 0.887	<0.001*
Number of children at home (2)	0.853	0.761, 0.956	0.73, 0.996	0.046*
Number of children at home (3)	0.684	0.483, 0.968	0.426, 1.098	0.117
Stress level (PHQ)	1.017	1.007, 1.027	1.003, 1.031	0.015*
Pre-existing psychiatric disease (Yes)	1.084	1.014, 1.159	0.989, 1.188	0.085
Pre-existing physical disease (Yes)	0.904	0.848, 0.963	0.829, 0.986	0.023*

**significance level (p<0.05)*
Global deviance: 4291.148
AIC: 4309.148
SBC: 4340.322
N=236
Degrees of freedom: 9
Residual degrees of freedom: 227

4. Sensitivity analyses of total health care costs (SHI-perspective)

Table 5: Sensitivity analyses total health care costs (SHI-perspective) considering 30% higher and lower intervention costs – excerpts from model estimations

	Exp(ß)	85 %-CI	95 %-CI	p-Value *(unadjusted)*
**Intervention costs +30%**				
Intercept	5086.529	4659.377, 5552.842	4513.940, 5731.751	<0.001*
Study group (IG)	1.109	1.041, 1.1801	1.018, 1.208	0.019*
**significance level (p<0.05)*<br>*Global deviance: 4265.477*<br>*AIC: 4283.477*<br>*SBC: 4314.651*<br>*N=236*<br>*Degrees of freedom: 9*<br>*Residual degrees of freedom: 227*				
Study group effect not significant after Bonferroni correction (new significance level p < 0.006).				
**Interventions costs -30%**				
Intercept	5080.417	4651.226, 5549.211	4505.151, 5729.139	<0.001*
Study group (IG)	1.084	1.017, 1.154	0.994, 1.181	0.068
**significance level (p<0.05)*<br>*Global deviance: 4264.075*<br>*AIC: 4282.075*<br>*SBC: 4313.249*<br>*N=236*<br>*Degrees of freedom: 9*<br>*Residual degrees of freedom: 227*				
Study group effect not significant after Bonferroni correction (new significance level p < 0.006).				

Table 6: Sensitivity analysis total health care costs (SHI-perspective) considering an extended study population (N=250)

	Exp(ß)	85 %-CI	95 %-CI	p-Value *(unadjusted)*
Intercept	5178.406	4758.965, 5634.815	4615.835, 5809.542	<0.001*
Study group (IG)	1.083	1.020, 1.150	0.998, 1.176	0.058
Number of children living in the same household (1)	0.800	0.750, 0.854	0.732, 0.874	<0.001*
Number of children living in the same household (2)	0.852	0.766, 0.948	0.737, 0.985	0.032*
Number of children living in the same household (3)	0.694	0.497, 0.967	0.441, 1.091	0.115
Stress level (PHQ)	1.015	1.005, 1.02	1.002, 1.027	0.023*
Pre-existing psychiatric disease (Yes)	1.095	1.029, 1.166	1.006, 1.192	0.037*
Pre-existing physical disease (Yes)	0.904	0.852, 0.959	0.834, 0.980	0.015*
**significance level (p<0.05)*<br>*Global deviance: 4512.166*<br>*AIC: 4530.166*<br>*SBC: 4561.859*<br>*N=250*<br>*Degrees of freedom: 9*<br>*Residual degrees of freedom: 241*				
Study group effect not significant after Bonferroni correction (new significance level p < 0.006).				

5. Multivariate analysis of overall physician and GP consultations

Table 7: Generalized additive model (negative binomial) – physician consultations (overall)

	**Exp(ß)**	**85 %-CI**	**95 %-CI**	**p-Wert** *(unadjusted)*
Intercept	31.237	27.696, 35.231	26.517, 36.797	<0.001*
Study group (IG)	0.859	0.768, 0.961	0.737, 1,000	0.052
Number of children at home (1)	0.836	0.937, 1.183	0.738, 0.948	0.006*
Number of children at home (2)	0.966	0.763, 0.917	0.788, 1.186	0.744
Number of children at home (3)	0.479	0.832, 1.123	0.248, 0.928	0.030*
Stress level (PHQ)	1.021	0.295, 0.779	1.003, 1.040	0.025*
Pre-existing physical disease (Yes)	0.799	1.008, 1.035	0.713, 0.895	<0.001*
Pre-existing psychiatric disease (Yes)	1.053	0.735, 0.869	0.898, 1.234	0.524
Study group (IG) : Pre-existing psychiatric disease (Yes)	1.407	1.187, 1.669	1.116, 1.775	0.004*
**significance level (p<0.05)*<br>*Global deviance: 1863.292*<br>*AIC: 1883.292*<br>*SBC: 1917.93*<br>*N=236*<br>*Degrees of freedom: 10*<br>*Residual degrees of freedom: 226*				

Table 8: Generalized additive model (negative binomial) – GP consultations

	**Exp(ß)**	**85 %-CI**	**95 %-CI**	**p-Wert** *(unadjusted)*
Intercept	4.301	2.747, 6.735	2.336, 7.921	<0.001*
Study group (IG)	1.224	1.014, 1.479	0.947, 1.584	0.124
Number of children at home (1)	0.628	0.513, 0.768	0.477, 0.826	0.001*
Number of children at home (2)	0.713	0.516, 0.984	0.459, 1.106	0.132
Number of children at home (3)	0.862	0.325, 2.287	0.228, 3.254	0.826
Educational level - Secondary school	1.381	0.905, 2.108	0.777, 2.456	0.273
Educational level - Technical college entrance qualification	1.943	1.245, 3.030	1.060, 3.559	0.033*
Educational level - University entrance qualification	1.164	0.776, 1.744	0.671, 2.019	0.590
Stress level (PHQ)	1.036	1.006, 1.067	0.995, 1.078	0.084
Pre-existing physical disease (Yes)	0.657	0.549, 0.785	0.515, 0.837	0.001*
**significance level (p<0.05)*<br>*Global deviance: 1273.441*<br>*AIC: 1295.411*<br>*SBC: 1333.497*<br>*N=235*<br>*Degrees of freedom: 11*<br>*Residual degrees of freedom: 224*				

6. Model diagnostics

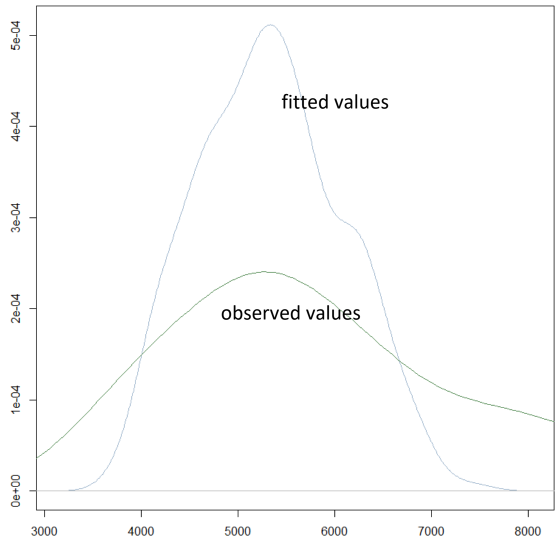

Figure 1: Density plot model estimation of total health care costs (SHI-perspective)

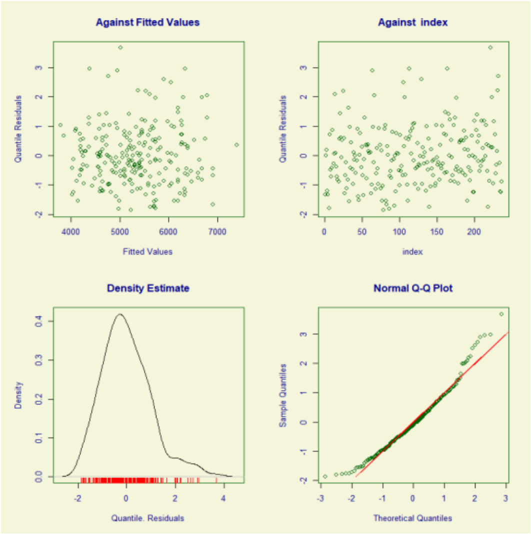

Figure 2: Model diagnostics model estimation of total health care costs (SHI-perspective)

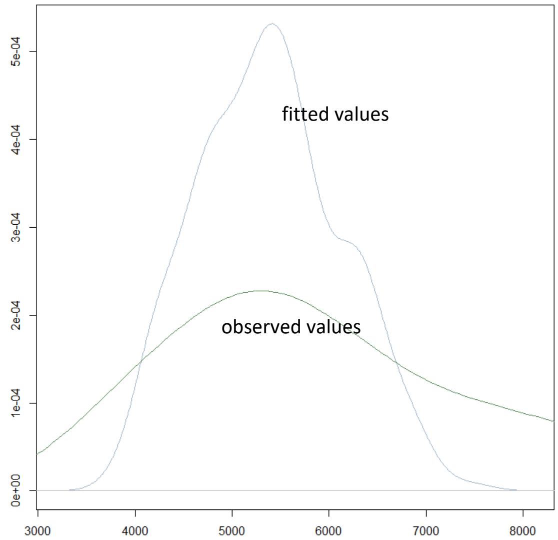

Figure 3: Density plot model estimation of total health care costs (societal perspective)

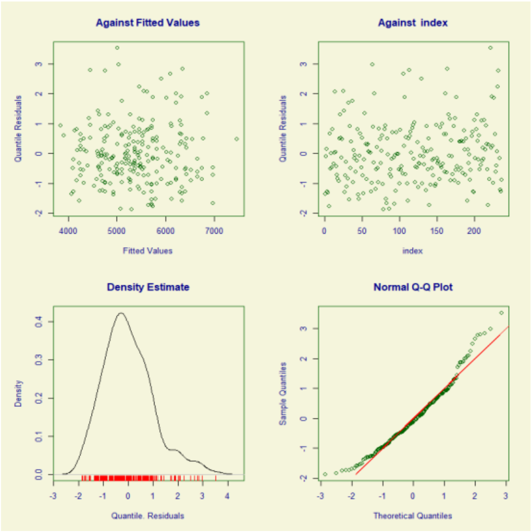

Figure 4: Model diagnostics model estimation of total health care costs (societal perspective)

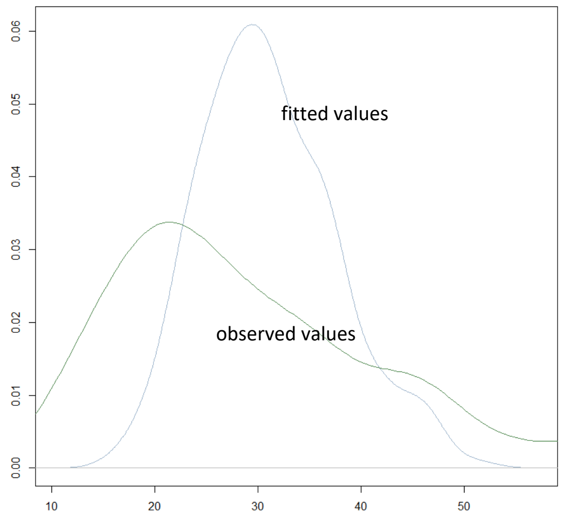

Figure 5: Density plot model estimation of physician consultations

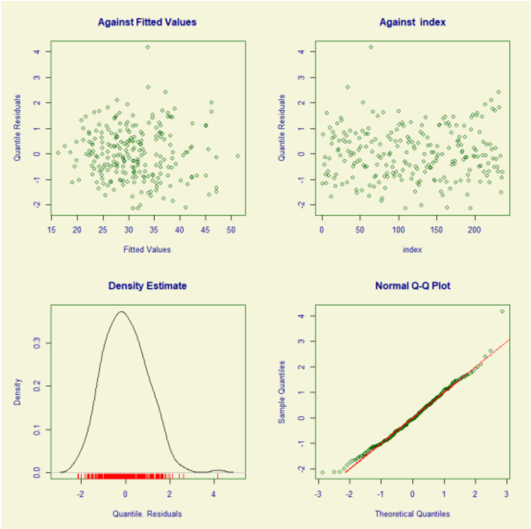

Figure 6: Model diagnostics model estimation of physician consultations

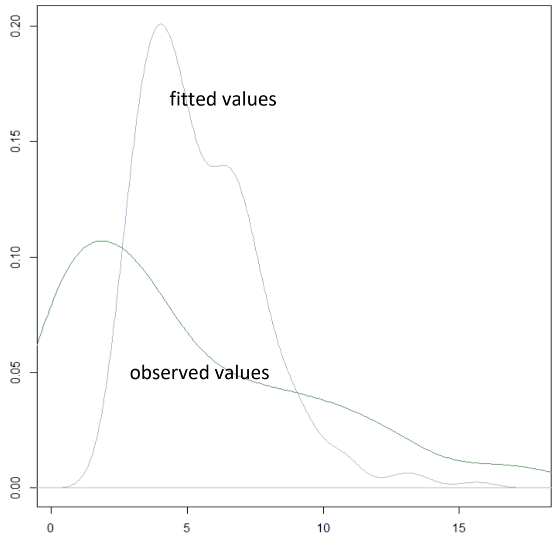

Figure 7: Density plot model estimation of general practitioner consultations

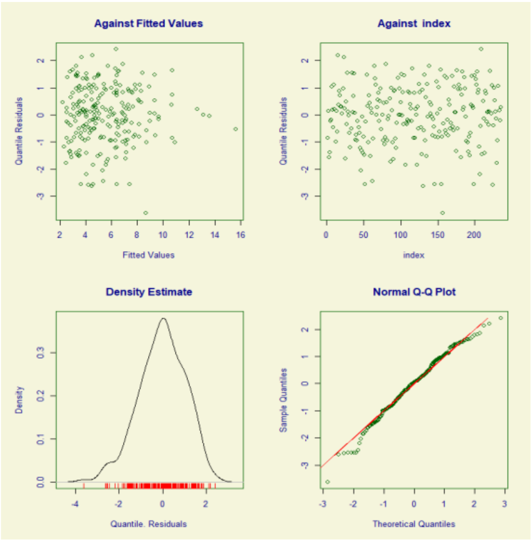

Figure 8: Model diagnostics model estimation of general practitioner consultations
